# High chromosomal variation in wild horn fly *Haematobia irritans* (Linnaeus) (Diptera, Muscidae) populations

**DOI:** 10.3897/CompCytogen.v9i1.8535

**Published:** 2015-02-05

**Authors:** Natalia S. Forneris, Gabriel Otero, Ana Pereyra, Gustavo Repetto, Alejandro Rabossi, Luis A. Quesada-Allué, Alicia L. Basso

**Affiliations:** 1 Cátedra de Genética, Facultad de Agronomía, Universidad de Buenos Aires, Av. San Martín 4453, C1417DSE CABA, Argentina; 2 Departamento de Producción Animal, Facultad de Agronomía, Universidad de Buenos Aires, Av. San Martín 4453, C1417DSE CABA, Argentina; 3 IIBBA-CONICET, Fundación Instituto Leloir and Dept. Biological Chemistry, Facultad de Ciencias Exactas y Naturales, Universidad de Buenos Aires, Patricias Argentinas 435, C1405BWE CABA, Argentina

**Keywords:** Karyotypes, genetic variability, population structure, B-chromosome, H-banding, evolution, chromosomal rearrangements

## Abstract

The horn fly, *Haematobia irritans* is an obligate haematophagous cosmopolitan insect pest. The first reports of attacks on livestock by *Haematobia irritans* in Argentina and Uruguay occurred in 1991, and since 1993 it is considered an economically important pest. Knowledge on the genetic characteristics of the horn fly increases our understanding of the phenotypes resistant to insecticides that repeatedly develop in these insects. The karyotype of *Haematobia irritans*, as previously described using flies from an inbred colony, shows a chromosome complement of 2n=10 without heterochromosomes (sex chromosomes). In this study, we analyze for the first time the chromosome structure and variation of four wild populations of *Haematobia irritans* recently established in the Southern Cone of South America, collected in Argentina and Uruguay. In these wild type populations, we confirmed and characterized the previously published “standard” karyotype of 2n=10 without sex chromosomes; however, surprisingly a supernumerary element, called B-chromosome, was found in about half of mitotic preparations. The existence of statistically significant karyotypic diversity was demonstrated through the application of orcein staining, C-banding and H-banding. This study represents the first discovery and characterization of horn fly karyotypes with 2n=11 (2n=10+B). All spermatocytes analyzed showed 5 chromosome bivalents, and therefore, 2n=10 without an extra chromosome. Study of mitotic divisions showed that some chromosomal rearrangements affecting karyotype structure are maintained as polymorphisms, and multiple correspondence analyses demonstrated that genetic variation was not associated with geographic distribution. Because it was never observed during male meiosis, we hypothesize that B-chromosome is preferentially transmitted by females and that it might be related to sex determination.

## Introduction

The economic importance of the horn fly, *Haematobia irritans* (Linnaeus, 1758) (Diptera: Muscidae) is based on its role as an obligate bloodsucking ectoparasite that plagues cattle around the world (Palmer and Bay 1981, Williams et al. 1985, Valério and Guimarães 1983). First reported in Argentina and Uruguay in 1991 (Luzuriaga et al. 1991, Carballo and Martínez 1991), it has been considered an economically important species in both countries, that can be found from the tropical North (22°02.22'S) to the temperate South (36°18.55'S) and between longitudes 64°33.67'W and 56°54'W (Anziani et al. 1993). Despite its economic importance, no information on the rate of eventual new invasions of this fly is available. In Argentina, the region called “Humid Pampa” has the most favourable conditions for the expansion of this insect and several generations per year, frequently produce sudden, extensive and damaging infestations of cattle (Bulman et al. 1999, Mancebo et al. 2001). Moreover, many local populations from Central and North Argentina demonstrated resistance to fenvalerate and pyrethroids (Torres et al. 1996, Sheppard and Torres 1998, Guglielmone et al. 2001), indicating significant changes in the descendants of the original invading stock that could make them even more invasive and destructive in the future. Insecticide resistance is one of the best examples of rapid microevolution found in nature (Silva et al. 2012) and has strong economical implications. Given the widespread presence of this pest in South Brazil and Paraguay (Sheppard and Torres 1998), and the prevalent cattle trade among these neighbouring countries, the horn fly population in Argentina is likely to be subjected to gene flow.

LaChance (1964) and Avancini and Weinzierl (1994) reported that *Haematobia irritans* showed a karyotype of 2n=10 composed of five pairs of chromosomes without a heteromorphic pair of sex chromosomes. As an insect source, all these researchers examined the same laboratory colony maintained at the USDA Livestock Insects Research Laboratory which, in turn, was derived from flies collected in the field at Kerrville, Texas (USA) and reared at least from 1962 on. Apart from *Haematobia irritans*, the only cyclorrhaphan Diptera having 2n=10 without a heteromorphic pair of chromosomes is *Muscina stabulans* (Fallen, 1817) (Parise et al. 1996, 2007). To our knowledge, no detailed cytological studies of wild horn flies are available.

Here we for the first time analyze wild populations of *Haematobia irritans* which have recently established in the Southern Cone of South America and exhibit considerable karyotypic diversity. The only related study is the analysis of genotypic variability in three horn fly populations from Brazil, Colombia and Dominican Republic, which was assessed by random amplification of polymorphic DNA (Gatto Brito et al. 2008). In addition, the mitochondrial genome of *Haematobia irritans* was studied by Oliveira et al. (2008).

## Materials and methods

### Ethics statement

This study was carried out on private lands, with kind permission from the landowners. For data regarding permissions for field collecting, please contact the corresponding author. Furthermore, we previously confirmed that no official permission was required for this field work, as it did not involve any endangered or protected species.

### Collection of adult horn flies and eggs

Sampling of adults from different populations of *Haematobia irritans* feeding on cattle was performed from 2004 to 2010 on private lands at the following main stations in Argentina: Ferreyra in Córdoba Province (31°56.67'S; 61°01.0'W); Trancas in Tucumán Province (26°21.66'S; 65°03.0'W); and Bolivar in Buenos Aires Province (35°50.0'S; 64°01.0'W). Sampling of adult *Haematobia irritans* from Uruguay was performed in Palmitas (33°25.0'S; 58°07.0'W). Under normal weather, populations of *Haematobia irritans* develop in these countries from early spring (October) to the beginning of fall (late March) (Guglielmone et al. 2001, 2002, Tarelli 2004), with the average degree of infestation depending on the climate. Samples showing high mortality when transferred from the field to the laboratory were discarded.

Adults were collected on livestock with a sweep net and transferred using positive phototropism to cages with rags soaked with 0.05% sodium citrate-added bovine blood as food source (Filiberti et al. 2009). Females were allowed to oviposit on pieces of cloth impregnated with 8.5 g/l NaCl solution for 12±1 h at 30 °C. After hatching, first-instar larvae were transferred to batches of urine-free bovine feces for feeding. Larval development took place in an environmental chamber at 29±1 °C. The life cycle of *Haematobia irritans* was partially described elsewhere (Basso et al. 2011).

### Cytology

Mitosis and meiosis were studied in neuroblasts of sub-esophageal ganglia of third-instar larvae and in spermatocytes of pharate and freshly eclosed adults respectively. Unfortunately, it was logistically impossible to simultaneously dissect brains and gonads in the same larvae.

### Preparation of ganglia

Ganglia were dissected in a drop of insect Ringer’s solution (Hayes 1953) and transferred first to 1% sodium citrate solution for 10–15 minutes, and then to methanol/acetic acid (3:1) for 40 seconds. After that, using a slide previously soaked in chilled methanol, each ganglion was transferred into a drop of freshly prepared and chilled 60% glacial acetic acid. The ganglion was then disaggregated with a pair of needles and a micropipette to disperse cells. After that, the slide was placed three times on a hot plate (75 °C) for 3 sec, air-dried, dehydrated, and stored at -20 °C before use.

### Preparation of testes

Testes of pharate adult males (120 h after puparium formation) and eclosed adults up to 36 h after emergence were used (Basso et al. 2011). The tissues were fixed and stained in a drop of lacto-acetic 2% orcein solution, covered with a cover slip and squashed 10 minutes later. Preparations with mitotic and meiotic metaphases were then sealed and stored at 6 °C before use.

### Chromosome banding and idiograms

Mitotic chromosome spreads from cerebral ganglia were C-banded using Ba(OH)_2_ treatment at 27–29 °C for 7 min and stained with 5% Giemsa Gurr (Merck, Germany) solution in phosphate buffer having pH=6.8 (Basso et al. 1995). H-banding was carried as described by Gatti et al (1976). The cytological preparations, described above, were re-hydrated with phosphate buffer having pH=7 (0.15 M NaCl, 0.03 M KCl and 0.01 M Na_2_HPO_4_) for 5 min. The slides were stained with 0.5 μg/ml Hoechst 33258 diluted in phosphate buffer during 10 min and then briefly rinsed with deionized water and air-dried. Mounting was performed in McIlvaine buffer with pH=7 (0.16 M dibasic sodium phosphate, 0.04 M sodium citrate). Preparations were kept in the dark during 24 hours before examination under a Zeiss Axioplan fluorescence microscope. Images were recorded with an Olympus DP72 digital camera, time exposure being manually adjusted. The relative chromosome length and centromere index were calculated after measurements taken both on preparations of ganglia and testes. The idiograms were drawn based on these measurements. Data were obtained from at least 10 metaphase plates per chromosome spread. Circa 1000 insects were dissected to obtain 287 individuals with good quality metaphases.

### Statistical analysis

Multiple Correspondence Analysis (MCA) is a method of factorial analysis that transforms a set of categorical or qualitative variables into a small number of orthogonal variables (Le Roux and Rouanet 2004). We grouped the individuals by seven karyotypic formulae. The variables that characterized the individuals were: the karyotypes classified into seven groups, the chromosome number (2n=10 or 2n=11), and the presence of satellites on each chromosome of the haploid karyotype (satellite [s1, s2, s3, s5] or absence of satellite). These 14 variables were used as active ones, while the geographical locations, i.e. Bolivar, Palmitas, Trancas and Ferreyra, were included as illustrative variables (Escofier and Pagés 1992, Lebart et al. 1995, 1996). Published data from Texas (LaChance 1964, Avancini and Weinzierl 1994) were also used in the analysis as illustrative individuals (n=24), i.e. as unique previous data to make comparisons. With the factorial axes from MCA, a hierarchical classification by Ward was imposed (Johnson and Wichern 1992). Data were processed using SAS software (2009) and SPAD3 (Lebart et al. 1996).

## Results

Chromosomes of four wild horn fly populations from Argentina and Uruguay were studied by cytological analysis of mitotic and meiotic metaphases. Brains of 232 third-instar larvae from three locations (Ferreyra, n=134; Bolivar, n=25; Palmitas, n=73) and testes of 55 individuals from three locations (Ferreyra, n=16; Trancas, n= 14; Palmitas, n=25) were analyzed.

Brain cell preparations from 72 to 96-hour third-instar larvae with empty guts showed mitotic pro-metaphases, metaphases and anaphases. Somatic pairing was observed in metaphase plates as reported for other dipterans (Stevens 1908, Metz 1916, Joyce et al. 2012). We distinguished two types of chromosome numbers within the studied samples: 2n=10 and 2n=11. All spermatocytes analyzed showed only 5 chromosome bivalents, i.e. 2n=10. No extra chromosome was found. Preparations from ovaries did not allow for chromosomal analysis since only interphase nuclei at the karyosome stage were present.

### Karyotype I: reference karyotype

Most specimens (n=150) were analyzed from Ferreyra. The most frequent chromosome number from this location was 2n=10, both on preparations from brains and testes. We arbitrarily defined the corresponding chromosome set as the reference karyotype (Karyotype I).

**Figure 1.**
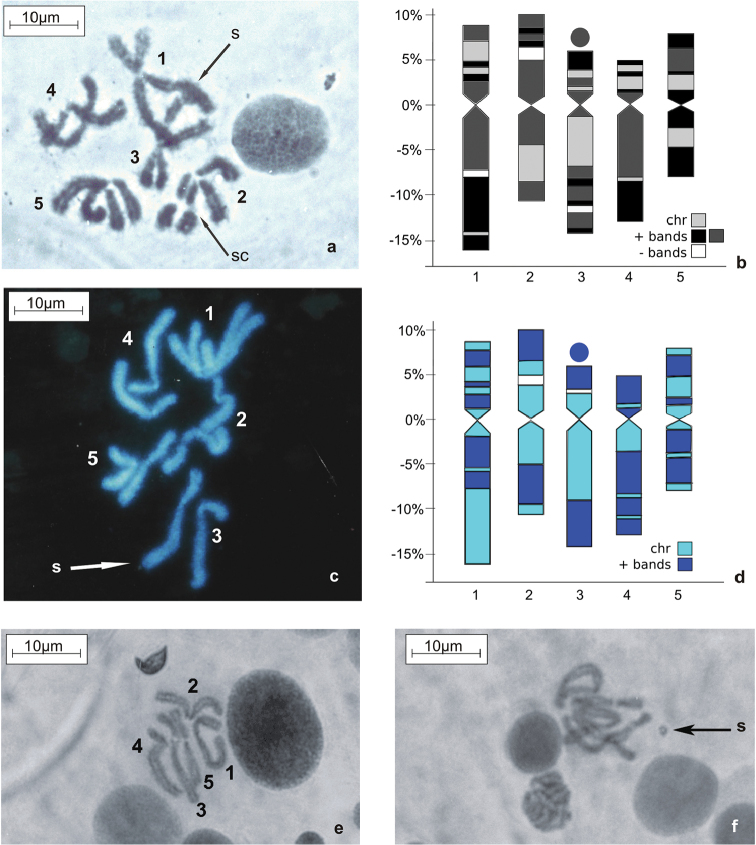
Reference karyotype of *Haematobia irritans* with 2n=10. **a** Giemsa stained C-banded mitotic metaphase from larval brain; arrow indicates satellite on chromosome 3, sc: secondary constriction **b** Idiogram of the C-banded haploid set **c** Hoechst 33258 stained H-banded mitotic metaphase from larval brain **d** Idiogram of the H-banded set **e–f** Orcein stained meiotic metaphases II from testis; both figures were observed in the same individual. **a**, **c**, **f** arrow indicates satellite on chromosome 3. sc: secondary constriction on chromosome 2.

Karyotype I showed two metacentric chromosomes (pairs 2 and 5) and three sub-metacentric chromosomes (pairs 1, 3 and 4) (Fig. 1a–f and Table 1). Chromosome 2 had a distinctive secondary constriction (Fig. 1a–b) which is the location of the nucleolar organizer (not shown), and chromosome 3 carried a satellite (Fig. 1a–b and Table 1). Sex chromosomes were not identified since no heterochromosomes (XY) could be distinguished from the autosomes. In male meiosis, a satellite on chromosome 3 was clearly observed in some spermatocytes (Fig. 1e–f).

**Table 1.** Frequency distribution of karyotypes within populations of *Haematobia irritans* from Argentina and Uruguay. sat: Chromosome carrying satellite; (a) grouping of karyotype formulae in the Multiple Correspondence Analysis.

A. Frequent karyotypes	Ferreyra n=150	Bolivar n=25	Trancas n=14	Palmitas n=98	(a)
Chromosome number 2n=10					
2M + 3SM					
Karyotype I (reference karyotype) 2M (2, 5) + 3SM (1, 3, 4); 3 sat	0.40	0.24	0	0.44	1
Karyotype II 1 sat	0.08	0	0.57	0.01	1
Karyotype III 2, 5 sat	0	0.24	0	0	1
Karyotype IV 3SM (2,3,4); 2 sat	0.02	0	0	0	2
1M + 4SM					
Karyotype V (inversion) 1M (2) + 4SM (1, 3, 4, 5); 1 sat	0.02	0	0.43	0.02	3
Chromosome number 2n = 11					
2M + 3SM + B					
Karyotype VI (translocation 1-5) 2M (1, 2) + 3SM (3, 4, 5) + B; 3 sat	0.32	0	0	0.43	4
1M + 4M/SM + B					
Karyotype VII (inversion) 1M (2) + 4M/SM (1, 3, 4, 5) + B; 2 sat	0.007	0.40	0	0	5
B. Rare karyotypes	Ferreyra n=150	Bolivar n=25	Trancas n=14	Palmitas n=98	
Chromosome number 2n=10					
5SM					
Karyotype VIII 5 SM (1, 2, 3, 4, 5)	0	0	0	0.01	6
1M/SM up to 5M/SM, chromosomal polymorphisms with complex rearrangements					
Karyotype IX (translocation 2-4) 1M (5) + 3SM (1, 3, 4) + 1M/SM (2); 2 sat	0.013	0	0	0	6
Karyotype X (translocation 2-3) 1M (5) + 2SM (1, 4)+ 2M/SM (2, 3); 2 sat	0.047	0	0	0	6
Karyotype XI 1M (2) + 2SM (3, 4) + 2M/SM (1, 5); 1, 5 sat	0	0	0	0.01	6
Karyotype XII 1M (2) + 2SM (3, 4) + 2M/SM (1, 5); 2, 5 sat	0.007	0	0	0.01	6
Karyotype XIII (inversion on chromosome 4) 2SM (1, 3) + 3M/SM (2, 4, 5)	0.013	0	0	0.02	6
Karyotype XIV (inversions) 1M (5) + 1SM (2) + 3M/SM (1, 3, 4)	0.007	0	0	0,01	6
Karyotype XV (translocations) 1SM (4) + 4M/SM (1, 2, 3, 5); 2 sat	0.02	0.04	0	0	6
Karyotype XVI 5 M/SM (1, 2, 3, 4, 5)	0.006	0	0	0.01	6
Chromosome number 2n = 11					
Mosaic specimens carrying nuclei with free or attached B chromosomes: formulae with heteromorphic pairs: SM ≠ SM+B					
Karyotype XVII (inversion on chromosome 4) 2M (2, 5) + 2SM (1, 3) + [1SM+B] (4); 2, 3 sat	0.02	0.04	0	0.01	7
Karyotype XVIII (inversion on chromosome 3) 1M(2) + 2SM(1, 4) + 1M/SM (5) + [1SM+B] (3); 2 sat	0.007	0	0	0.01	7
Karyotype XIX (inversions on chromosomes 3, 4) 2M (2, 5) + 1SM(1)+ [2SM+B] (3, 4); 2 sat	0.013	0.04	0	0.01	7
Total	1	1	1	1	

C-banding of karyotype I allowed for detection of satellites (Fig. 1a). The idiogram of C-banded haploid karyotype I is shown on Fig. 1b. Chromosome 1 carried a wide C-block on the long arm and one distal band on the short arm. Chromosome 2 showed a conspicuous C-negative band on one arm that marked a secondary constriction. The long arm of chromosome 3 showed three narrow distal marks and a secondary constriction followed by a conspicuous satellite (Fig. 1b). Chromosome 4 carried a wide C-block nearly occupying the entire long arm. Chromosome 5 showed a narrow interstitial positive band within a distinct C-banded arm while the other arm revealed no bands. All chromosomes showed pericentromeric C-bands at least in one arm (Fig. 1a–b).

H-banded karyotype I is documented on Fig. 1c. The idiogram of the H-banded haploid set is shown on Fig. 1d. Prominent H-bands were observed in all the chromosomes, and apart from chromosome 4, H-bands did not mark the centromeres. A secondary constriction on chromosome 2 was marked by a gap, i.e. a negative H-band. Chromosome 3 carried positive H-bands on the satellite and the distal regions of both arms. Chromosomes 4 and 5 had large H-bands; chromosome 4 carried a centromeric H-band on the short arm (Fig. 1c–d).

### Chromosomal rearrangements and heteromorphisms

**Figure 2.**
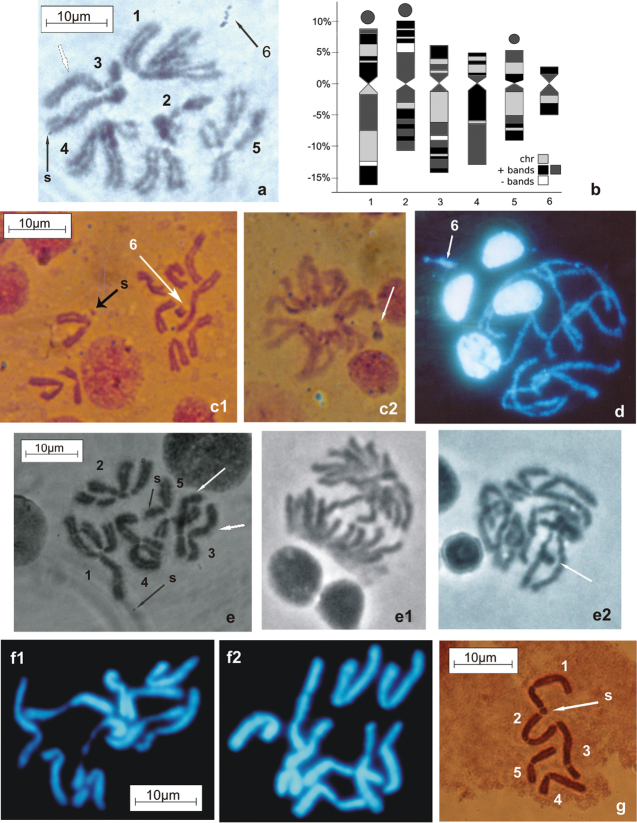
Chromosome variants in *Haematobia irritans*. **a–f** mitotic plates from larval brain cells. **a** Giemsa C-banded metaphase plate with 2n=11 and karyotype VI; black arrow indicates B-chromosome, white arrow indicates relocation of secondary constriction on the curved long arm of one homologue of pair 3, “s” indicates satellite on chromosome 3 **b** Composite C-banding idiogram showing some chromosome variants found in different specimens **c** Giemsa C-banded metaphases with 2n=11 found in the same larva with karyotype VII; white arrow indicates B-chromosome, black arrow indicates satellite on metacentric chromosome 2 **d** Hoechst 33258 stained prometaphase with 2n=11 carrying complex rearrangements; white arrow indicates B-chromosome **e** Giemsa stained C-banded metaphase with 2n=10 and heteromorphic pairs 3 and 4; black arrows indicate satellites on chromosomes 1 and 5, white arrows indicate both long arms of pair 3 with one of them carrying an attached B-chromosome **e1** anaphase with a bridge **e2** metaphase from the same specimen as in **e** and **e1**; white arrow indicates rearrangement in one of the homologues of pair 3, black arrow on pair 4 **f** Hoechst 33258 stained metaphases with 2n=10 and karyotype XV carrying complex rearrangements **g** Orcein stained meiotic metaphase II from testis with karyotype V; white arrow indicates satellite on chromosome 1.

Using karyotype I as the reference one, somatic chromosomal polymorphisms affecting chromosome number and/or morphology were detected. Cytological preparations with 2n=11 but without a heteromorphic chromosome pair were selected. This karyotype is formed due to presence of an acrocentric chromosome carrying a small C-positive short arm (Fig. 2a, b, c1, c2, d and Table 1). A summary of variants is represented in the composite C-banding idiogram (Fig. 2b). Chromosome 2 always carried a secondary constriction in all individuals from different populations (see below). Additionally, C- and H-banding revealed variation in the number and position of the bands and in the presence of satellites on chromosomes 1, 2, and 5; no satellite was found on chromosome 4 (n=287) (Fig. 2c1, e, g and 3a). Major changes in C-heterochromatin distribution affected pairs 1, 2 and 5 (Fig. 1b and 2b). The polymorphic long arm of chromosome 3 showed a curved shape on one homologue, lack of pairing and a change in the position of secondary constriction (Fig. 2b, e, e2). A variant of chromosome 4 showed change in C-heterochromatin distribution on the long arm: one of the homologs exhibited the darkest band on the long inverted arm, leading to lack of pairing (Fig. 1b, 2b, e). In the same specimen, a chromosomal bridge (2e1) as well as another metaphase plate (Fig. 2e2) with polymorphic pairs 3 and 4, add further evidence for these rearrangements, as they could arise from an inversion. A submetacentric variant of chromosome 5 was revealed by C-banding (Fig. 2a, b and e). The extra small chromosome was positively H-banded (Fig. 2d). Unpaired chromosomal segments were recognized in two metaphases of the same specimen (Fig. 2f1, f2), indicating banding heteromorphisms and structural polymorphisms.

### Frequent and rare karyotypes

**Figure 3.**
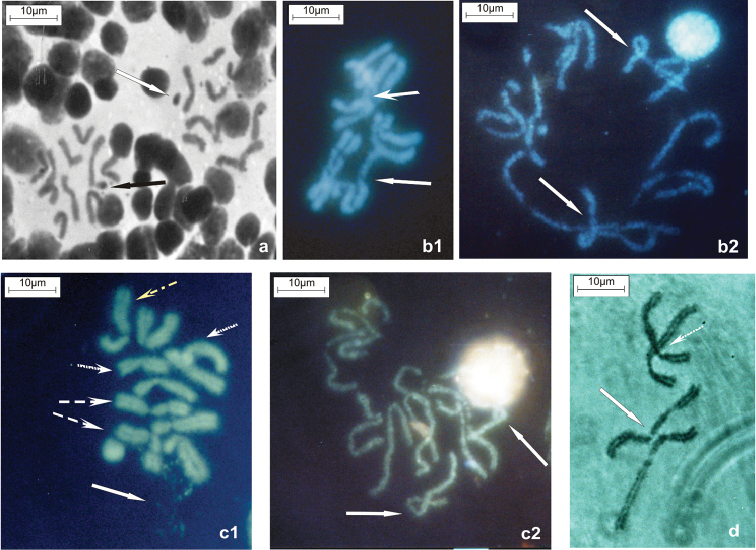
Chromosome variants in *Haematobia irritans*. Mitotic plates from larval brain cells. **a** Two C-banded metaphases with karyotype XVIII, one with 2n=11 (2n=10+B) and the other with 2n=10 with free and attached B-chromosomes (white and black arrows respectively) **b1** and **b2** Hoechst 33258 stained metaphase and prometaphase of the same specimen with karyotype IV; arrows indicate rearranged pairs 1 and 2 **c** Hoechst 33258 stained metaphase and prometaphase **c1** heteromorphisms of pairs 1 (dashed yellow arrow), 4 and 5 (dotted arrows) and the nucleolus close to chromosome 2 (dashed white arrow), expression of ribosomal DNA located on secondary constriction on chromosome 2 (white arrow) **c2** H-banded karyotype with heteromorphisms; arrows indicate rearrangements on prometaphase chromosomes **d** C-banded incomplete metaphase with chromosomal pairs 1 and 3 (dotted arrow); white arrow indicates pair 1 carrying heterozygous inversion.

We identified 19 main chromosomal profiles in Argentina and Uruguay (Table 1), through differences in the chromosome banding (heteromorphisms), chromosome rearrangements (polymorphisms) and chromosome number (Fig. 2a, c–g and 3a–d); frequency distribution of these profiles within each population was calculated. These profiles were classified in seven chromosomal formulae (Table 1). Table 1A groups the most frequent chromosomal formulae (≥0.150) with the exception of formula 1M + 4SM (see below), whereas rare karyotypes are shown in Table 1B.

Karyotypes composed of two metacentric and three submetacentric chromosomes (formula 2M + 3SM) are represented by variants I, II, III (Table 1A, column “a” Group 1) and IV (Table 1A, column “a” Group 2). Karyotype II is similar to karyotype I but with a satellite on chromosome 1. Karyotype III showed satellites on chromosomes 2 and 5. Karyotype IV has undergone a significant change that gave rise to metacentric chromosome 1 and submetacentric chromosome 2 (Fig. 3b1 and b2) and thus we used it in our Multiple Correspondence Analysis (Table 1, column “a” Group 2). Karyotype V has one metacentric (chromosome 2) and four submetacentric chromosomes, i.e. 1M + 4SM (Table 1, Group 3); chromosome 1 carries a satellite (Fig. 2g). This karyotype is dominant in Trancas population.

Karyotypes with 2n=11 (2n=10+B) and formula 2M + 3SM + B correspond to karyotype VI (Fig. 2a) (Table 1A, Group 4),whereas those with formula 1M + 4M/SM + B carrying four polymorphic pairs formed by one metacentric and one submetacentric chromosome correspond to karyotype VII (Fig. 2c1 and c2) (Table 1A, column “a” Group 5). Karyotype VI appeared to be dominant in our samples from Ferreyra and Palmitas, whereas karyotype VII was dominant in Bolivar.

Rare karyotypes (Table 1B) were not found in the Trancas population. Formula 5SM corresponds to karyotype VIII (2n=10), composed of five pairs of submetacentric chromosomes (not shown). Only one specimen of that kind was found in the Palmitas population (Table 1B column “a” Group 6). The sixth formula, 1M/SM to 5M/SM (2n=10) includes eight very scarce karyotypes (IX to XVI) from Ferreyra and Palmitas, carrying 1 to 5 polymorphic pairs composed of one metacentric and one submetacentric chromosome (Table 1B, Group 6). C- and H- bandings provide strong evidence for complex rearrangements along with lack of somatic chromosomal pairing (Fig. 2d, 2f1, 2f2, 3b1, 3b2, 3c1, 3c2). Rare karyotypes XVII, XVIII and XIX with formula SM≠SM + B (Table 1B, column “a” Group 7) include mosaic insects carrying nuclei with 2n=10 and 2n=11 and having polymorphic pairs of chromosomes (Fig. 3a); the extra chromosome is free or attached to one of the homologs of pair 3 or pair 4 (Table 1B, Group 7) (Fig. 2e, e1, e2).

### Population structure

Within the Ferreyra population, karyotype I (2n=10) and karyotype VI (2n=11) were present in 40% and 32% of the insects respectively (Table 1). Due to the fact that the sample size of this population was large (n=150), we detected specimens with 16 different karyotypes (Table 1). Insects with 2n=10 and chromosome variations represented 23.3% within this population; those included karyotypes II, IV (Fig. 3b1, b2 and Table 1) and all the series of karyotypes from 1M/SM up to 5M/SM (group 6). All the karyotypes with 2n=11 (Table 1, groups 4, 5 and 7) were present in 36.7% of Ferreyra specimens. However, the extra chromosome was never observed in preparations from the testes.

Within the Bolivar population, 52% flies had 2n=10. The frequency of karyotype I was 24%. Interestingly, karyotype III with the same frequency and satellites on chromosome 2 was unique to this population (Table 1). Additionally, karyotype VII with 2n=11 (Fig. 2a, c1, c2, d, 3a and Table 1) was present in 40% of the insects within Bolivar sample.

In the Trancas population, only a small sample was studied on the basis of male meiosis. All the insects had karyotype II (2M + 3SM) or karyotype V (1M + 4SM) (Fig. 2g and Table 1). Both karyotypes showed a satellite on chromosome 1, but they differed in the morphology of chromosome 5, which appeared to be metacentric in karyotype II but submetacentric in karyotype V.

In the Palmitas population, karyotypes I (2n=10) (44%) and VI (2n=11) (43%) were dominant. Although 56% insects carried rearrangements, flies having 2n=11 prevailed (46%; Table 1). Karyotypes II, V, VIII, IX, X, XII and XIV also had structural rearrangements (10%). This was the only population where an individual carrying karyotype VIII with five submetacentric pairs (formula 5SM) was found. Karyotype II was uncommon in Palmitas but frequent in Trancas (Table 1).

**Figure 4.**
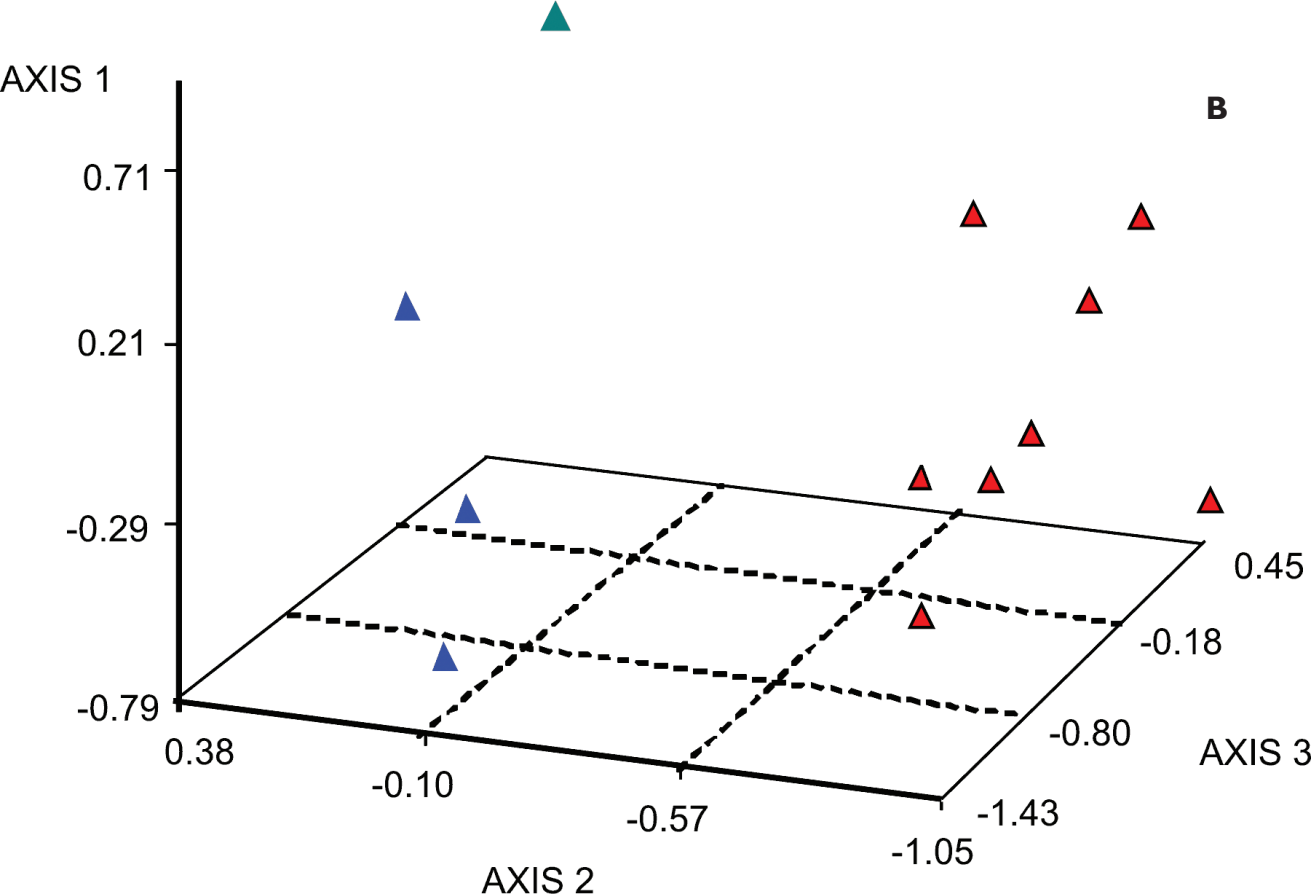
Clustering and spatial distribution of *Haematobia irritans*. **a** The most significant discrimination is obtained by the first three axes: karyotypes, satellites and zygotic number of chromosomes, and therefore the three clusters are the most representative. Texas individuals (LaChance 1964, Avancini and Wienzierl 1994) were also included **b** Spatial distribution of individuals; clusters 1 to 3 and Texas individuals are shown in blue, red, green and black respectively.

MCA produced 14 axes from 14 nominal variables (see Materials and Methods). From these, we have chosen seven factors that explained 92.25% of the whole variation. Graphical displays constructed from cluster analysis obtained from those factors, were used to summarize proximities between the specimens and to show associations between the categorical variables. The dendrogram (Fig. 4a) showed the hierarchical classification, where the greatest discrimination was obtained by the first three factors: karyotypes, satellites and zygotic chromosome number. Therefore the three clusters that grouped similar individuals were the most representative. As expected, the factorial analysis has clearly shown that localities were not a discriminating factor.

Cluster 1 (n=141) is characterized by individuals from groups 1 and 3 (Table 1A, column “a”), with 2n=10, and satellites on chromosome 1 (s1), or on chromosome 2, 3 or 5. This cluster grouped all Trancas individuals, 50% of Ferreyra individuals and 47% of Palmitas individuals. Neither specimens from groups 2, 4, 5, 6 and 7 nor those with 2n=11 were found in this cluster (Table 1A, column “a”).

Cluster 2 (n=56) is characterized by all individuals of groups 2, 5, 6 and 7 (Table 1, column “a”); those with 2n=10 predominated. All insects with satellites on chromosomes 2 or 5 as well as those without satellites were found in this cluster. There were very few individuals of group 1 (10.71%) and no individuals of group 4 (Table 1, column “a”). Cluster 2 grouped 76%, 10% and 18% of Bolivar, Palmitas and Ferreyra individuals respectively.

Cluster 3 (n=90) is characterized by individuals with 2n=11, of group 4 and with a satellite on chromosome 3; it is composed by 32% of Ferreyra individuals and 43% of Palmitas individuals (Table 1A, column “a”).

The specimens were represented in three-dimensional graphical displays constructed using the first three factorial axes (Fig. 4b). Data on the flies from Texas (LaChance 1964, Avancini and Weinzierl 1994) are also given (in black); the spatial distribution of all individuals (Fig. 4b) showed them as a part of cluster 2 (in red). The clusters well differ from each other; all individuals in cluster 3 have identical karyotypes.

## Discussion

This study is the first chromosomal analysis of recently established wild populations of the horn fly, originally introduced in Brazil in 1983, in the Southern Cone of South America. We believe that the karyotype study provides a basic tool for understanding the population dynamics. Unfortunately, this research is limited by the lack of a full-cycle laboratory rearing technique of the horn fly.

### Chromosomal rearrangements

We here document chromosomal rearrangements that affect the chromosome number and morphology. We also confirm that the main chromosome number in *Haematobia irritans* is 2n=2x=10. For the first time we show the existence of an extra chromosome in three populations, resulting in the presence of chromosome sets with 2n=11 (2n=10+B).

Our study showed that the main chromosomal formula for the karyotypes found in Ferreyra, Palmitas, Trancas and Bolivar was 2M + 3SM. Variation found in the populations from Ferreyra, Palmitas and Bolivar (Table 1), involved some complex chromosomal rearrangements such as those in karyotypes VIII to XVI (Table 1B) and mosaic specimens such as those with karyotypes XVII to XIX carrying nuclei with 10 and 11 chromosomes. Within the best-analyzed populations, i.e. Ferreyra and Palmitas, individuals with 5M/SM or 5SM were found within all clusters (Fig. 4a, b). This karyotype diversity was previously reported neither by LaChance (1964) nor by Avancini and Weinzierl (1994). LaChance (1964) studied chromosome sets of the horn fly on the preparations from larval brain cells (n=18) or testes (n=11) and described a unique karyotype of *Haematobia irritans* with 2n=10 composed by five pairs of autosomes (4M + 1 SM) without a distinct secondary constriction. Thirty years later, Avancini and Weinzierl (1994) described individuals from the same colony, but with only one karyotype 2n=10, composed by five pairs of autosomes (3M +2 SM) based on the analysis of “ten adult testis cells in meiotic metaphase” or “a few larval brain cells”; those authors found that submetacentric chromosome 3 seemed to carry the nucleolar organizer in the pericentromeric region. However, we found that chromosome 2 carried a secondary constriction that denotes the location of the nucleolar organizer (not shown) and can reveal a satellite during the cell cycle. Mobility of the satellites between different chromosomes can be explained either by transposition or fragmentation of the nucleolar organizer.

### Origin and transmission of the small extra chromosome

Extra chromosomes were found within karyotypes from Ferreyra, Bolivar and Palmitas, but we could not determine whether it was the same B-chromosome in all cases. A B-chromosome might be derived from a fragmented autosome or a sex chromosome, i.e. a centric fragment derived from amplification of the paracentromeric region (Fig. 2b–e, 3a, Table 1). It could originate by inversions that require at least two breaks on the same chromosome.The small chromosome could arise by a pericentric inversion taking place close to one inessential chromosomal end (3b1, 3b2, 3d). When two crossing-overs involving three chromatids take place, i.e. one within the inverted region and the other within the proximal region, a bridge gives origin to small chromosomes (Fig. 2b, e, e1, e2).

Lampe et al. (1998, 2001) reported the presence of transposons in *Haematobia irritans*, and *HIMAR1* is one of the only two known active mariner elements (Lampe et al. 1998). Heterochromatic B-chromosomes, usually revealed by C-banding, typically originate from satellite blocks of repeated DNA sequences which vary in type, kind of repeat, and copy number (Franks et al. 1996), as a result of unequal crossing-over and reduced recombination (Bigot et al. 1990, Charlesworth and Sniegowski 1994). Camacho et al. (2000) proposed that B-chromosomes might be amalgamations of transposable DNA. As indicated in Table 1, rearrangements affecting chromosomes 3 or 4 were associated with B-chromosomes (Fig. 2e, e2; autosome 3, Fig. 3a). Analyses of C-banding (Fig. 1b, 2a, b, c1, c2, e, e1, e2) and H-banding patterns in brain larval cells (Fig. 1c, d, 2d–f1, f2) at the population level, suggested that the extra small chromosomes might originate through breaks within chromosome 3 or chromosome 4.

Population frequencies of B-chromosomes result from a balance between their transmission rates and their effects on host fitness (Basso and Lifschitz 1995, Camacho et al. 2000). Our observations suggest that a free B-chromosome is only transmitted via female meiosis. Differential transmission of the B-chromosome was cytogenetically studied in *Ceratitis capitata* (Wiedemann, 1824) (Basso and Lifschitz 1995), where 63 progenies from reciprocal crossings were studied and the B-chromosome observed in both sexes, in somatic cells (cerebral ganglia tissue) as a free chromosome, or terminally attached to the long arm of the X-chromosome, appearing as a larger X. Males transmit both free Bs and large X-chromosomes to their progeny. Females of *Ceratitis capitata* transmit the large Xs to their progeny, at a higher rate than the standard Xs, suggesting either differential fate of the oocytes or a preferential co-orientation during the first meiotic division. A similar phenomenon that involved preferential survival of spermatozoa bearing Bs was also described in this species by Manso and Lifschitz (1986).

In our studies, the extra chromosomes might be associated with sex determination as the frequencies of individuals with 2n=11 in Ferreyra, Bolivar and Palmitas were 0.367, 0.48 and 0.46 respectively. The extra chromosome was observed in around 50% of larval brains and was not observed in preparations from testes, further suggesting that the B-chromosome is restricted to females. We hypothesize that this B-chromosome is preferentially transmitted by females, and that it might be related to sex determination because it was never observed during male meiosis. Sex chromosomes were previously proposed as ancestors of the Bs (Hewitt 1973). At the parsimonious hypothesis, homomorphic chromosomes may have sex determination factor like in *Aedes aegypti* (Hall et al. 2014). In spite of the difficulties to demonstrate B-chromosomes in gonad tissues, we believe that extra chromosomes behave in a similar way that those reported in *Ceratitis capitata* (Basso and Lifschitz 1995), because they are transmitted from parents to the offspring.

### Rearrangements, hybridization and polymorphisms

Although a direct investigation of demographic structure is difficult in natural conditions (Roderick 1996), study of the genetic structure of wild horn fly populations that is affected by mutations, migration or gene flow, selection and genetic drift, is a basic tool to understand population dynamics and insecticide resistance that must be complemented by DNA sequencing studies.

MCA generated three clusters: cluster 1 grouped specimens with 2n=10 from all four populations; cluster 2 grouped rare karyotypes with 2n=10 and 2n=11 as well as insects from Texas (Fig. 4a–b); cluster 3 grouped insects with 2n=11 from Ferreyra and Palmitas. Since karyotypes from Texas were respectively reported 50 and 20 years ago (LaChance 1964, Avancini and Weinzierl 1994), loss of chromosome variants was probably due to inbreeding, thus both karyotypes were grouped in cluster 2. We did not find the karyotypes from Kerrville within our populations. However, other less frequent chromosome sets carrying heteromorphic M/SM pairs, grouped in the same cluster together with Texas karyotypes. Moreover, it is significant that the correspondence analysis revealed that localities did not discriminate between populations, apart from the chromosome number and karyotype structure (Fig. 4a–b). Since up to 1200 generations of the horn fly during the last 50 years have occurred, metacentric chromosomes that were reported by LaChance (1964) and Avancini and Weinzierl (1994), may have been transformed into submetacentric ones. Inversions which reduced interchromosomal recombination and also maintained the polymorphism, are likely to have been responsible for this significant change. On the other hand, hybridization produced further recombination between the different variants.

Abundant karyotypes were analyzed in two locations, Ferreyra in Argentina and Palmitas in Uruguay. Resilience of genomes to massive introgression through hybridization, can allow for rapid adaptive response to anthropogenic selection (Clarkson et al. 2014). Thus, our results explain repeated appearance of resistant phenotypes in *Haematobia irritans*.

## Conclusions

We confirmed the chromosome set with 2n=10 as the reference karyotype in wild populations of *Haematobia irritans* invading the Southern Cone of South America. Karyotypic variants were characterized for the first time; half of these variants were 2n=11 due to the presence of a B-chromosome.

The B-chromosome was observed only in mitotic divisions, mainly as a free acrocentric chromosome.

Horn fly control will highly benefit from genetic studies focusing on the understanding of sex determining mechanisms, which are necessary to design appropriate control strategies, as related to the adaptation of these insects to control measures such as insecticides.

## References

[B1] Anziani A Guglielmone A Vogel A Mangold A Dotti M Volpogni E (1993) Control de *Haematobia irritans* en vacas lecheras utilizando bolsas autoaplicadoras con coumafós. Revista de MedicinaVeterinaria 74: 185–188.

[B2] Avancini R Weinzierl R (1994) Karyotype of the Horn Fly, *Haematobia irritans* (L.) (Diptera, Muscidae). Cytologia 59: 269–272. doi: 10.1508/cytologia.59.269

[B3] Basso AL Lifschitz E (1995) Size polymorphism of the X-chromosome due to attachment of the B-chromosome in the Medfly, *Ceratitis capitata* (Wied). Brazilian Journal of Genetics 18: 165–171.

[B4] Basso AL Forneris N Filiberti A Argaraña C Rabossi A Quesada-Allué LA (2011) Metamorphosis and gonad maturation in the horn fly *Haematobia irritans*. Journal of Insect Science 11: 174–185. doi: 10.1673/031.011.17401 22957976 10.1673/031.011.17401 PMC3465833

[B5] Basso AL Lifschitz E Manso F (1995) Determination of intraspecific variation in sex heterochromatin of *Ceratitis capitata* (Wied.) by C-banding. Cytobios 83: 237–244.

[B6] Bigot Y Hamelin M Periquet G (1990) Heterochromatin condensation and evolution of unique satellite-DNA families in two parasitic wasp species *Diadromus pulchellus* and *Eupelmus vuilleti* (Hymenoptera). Molecular Biology and Evolution 7(4): 351–364. 2385173 10.1093/oxfordjournals.molbev.a040611

[B7] Bulman G Lamberti J Mancebo O Guglielmone A Margueritte J Filippi J (1999) *Haematobia irritans* y su control en Argentina: pasado, presente y futuro. Revista Therios 28: 188–198.

[B8] Camacho J Sharbel T Beukeboom L (2000) B-chromosomes evolution. Philosophical Transactions of the Royal Society of London. Series B. Biological Sciences 355: 163–178. doi: 10.1098/rstb.2000.0556 10724453 10.1098/rstb.2000.0556 PMC1692730

[B9] Carballo M Martínez M (1991) Hallazgo de *Haematobia irritans* en Uruguay. Veterinaria (Montevideo) 27: 20–21.

[B10] Clarkson CS Weetman D Essandoh J Yawson A Maslen G Manske M Field S Webster M Antao T MacInnis B Kwiatkovsky D Donnely M (2014) Adaptive introgression between *Anopheles* sibling species eliminates a major genomic island but not reproductive isolation. Nature Communications 5: 4248. doi: 10.1038/ncomms5248 10.1038/ncomms5248 PMC4086683 24963649

[B11] Charlesworth B Sniegowski W (1994) The evolutionary dynamics of repetitive DNA in eukaryotes. Nature 371: 215–220. doi: 10.1038/371215a0 8078581 10.1038/371215a0

[B12] Escofier B Pagés J (1992) Análisis factoriales simples y múltiples; objetivos, métodos e interpretación. Servicio Editorial de la Universidad del País Vasco. 2da. Ed, Paris, 119 pp.

[B13] Filiberti A Rabossi A Argaraña C Quesada-Allué LA (2009) Evaluation of Phloxine B as photoinsecticide on Immature Stages of the Horn Fly, *Haematobia irritans* (Diptera: Muscidae). Australian Journal of Entomology 48: 72–77. doi: 10.1111/j.1440-6055.2008.00686.x

[B14] Franks T Houben A Leach C Timmis J (1996) Chromosoma 105: 223–230. doi: 10.1007/BF02528770 8854881 10.1007/BF02528770

[B15] Gatti M Pimpinelli S Santini G (1976) Characterization of *Drosophila* heterochromatin. I. Staining and decondensation with Hoechst 33258 and quinacrine. Chromosoma 57: 351–375. doi: 10.1007/BF00332160 63358 10.1007/BF00332160

[B16] Gatto Brito L Regitano L Huacca M Carrilho M Paes MJ Moya-Borja G (2008) Genotype characterization of the *Haematobia irritans* (Diptera: Muscidae) from Brazil, Dominican Republic and Colombia based on RAPD analysis. Revista Brasilera de Veterinaria 17: 179–184. 10.1590/s1984-29612008000400002 19265574

[B17] Guglielmone A Castelli M Volpogni M Medeus P Anziani O Mangold A (2001) Comparación de la concentración letal 50 de Diazinón y cipermetrina para *Haematobia irritans* resistentes (Diptera: Muscidae) entre áreas de producción de leche o de carne en Santa Fe y Entre Ríos, Argentina. Revista de Medicina Veterinaria (Bs.As.) 82: 209–211.

[B18] Guglielmone A Castelli M Volpogni M Anziani O Mangold A (2002) Dynamics of cypermethrine resistance in the field in the horn fly *Haematobia irritans*. Medical and Veterinary Entomology 16: 310–315. doi: 10.1046/j.1365-2915.2002.00380.x 12243232 10.1046/j.1365-2915.2002.00380.x

[B19] Hall AB Timoshevskiy VA Sharakhova MV Jiang X Basu S Andersdon MAE Hu W Sharakhov IV Adelman ZN Tu Z (2014) Insight into the preservation of the homomorphic sex-determining chromosome of *Aedes aegypti* from the discovery of a male-biased gene tightly linked to the M-locus. Genome Biology Evolution 6: 179–191. doi: 10.1093/gbe/evu002 24398378 10.1093/gbe/evu002 PMC3914700

[B20] Hayes R (1953) Determination of a physiological saline solution for *Aedes aegypti* (L.). Journal of Economic Entomology 46: 1–7. doi: 10.1093/jee/46.4.624

[B21] Hewitt G (1973) The integration of supernumerary chromosomes into the orthopteran genome. Cold Spring Harbor Symposium. Quantitative Biology 38: 183–194. doi: 10.1101/SQB.1974.038.01.022 4524756 10.1101/sqb.1974.038.01.022

[B22] Johnson R Wichern D (1992) Applied Multivariate Statistical Analysis, Prentice-Hall, Third Edition Englewood Cliffs, New Jersey.

[B23] Joyce EF Williams BR Xie T Wu C-t (2012) Identification of genes that promote or antagonize somatic homolog pairing using a high-throughput FISH-based screen. PLoS Genetics 8(5): e1002667. doi: 10.1371/journal.pgen.1002667 10.1371/journal.pgen.1002667 PMC3349724 22589731

[B24] LaChance LE (1964) Chromosome studies in three species of Diptera (Muscidae and Hypodermatidae). Annals of the Entomological Society of America 57: 69–73. doi: 10.1093/aesa/57.1.69

[B25] Lampe DJ Grant TE Robertson HM (1998) Factors affecting transposition of the *Himar1* mariner transposon *in vitro*. Genetics 149: 179–187. 9584095 10.1093/genetics/149.1.179 PMC1460121

[B26] Lampe DJ Walden K Robertson HM (2001) Loss of transposase-DNA interaction may underlie the divergence of *mariner* family transposable elements and the ability of more than one mariner to occupy the same genome. Molecular Biology and Evolution 18: 954–961. doi: 10.1093/oxfordjournals.molbev.a003896 11371583 10.1093/oxfordjournals.molbev.a003896

[B27] Lebart L Morineau A Piron M (1995) Statistique exploratoire multidimensionnelle. Dunod, Paris.

[B28] Lebart L Morineau A Lambert T Pleuvret P (1996) Manuel de référence. SPAD Version 3. CISIA, Saint- Mandé, France.

[B29] Le Roux B Rouanet H (2004) Geometric Data Analysis, From Correspondence Analysis to Structured Data Analysis. Kluwer, Dordrecht, 180 pp.

[B30] Luzuriaga R Eddi C Caracostantógolo J Botto E Pereira J (1991) Diagnóstico de parasitación con *Haematobia irritans* (L) en bovinos de Misiones, República Argentina. Revista de Medicina Veterinaria (Bs. As) 72: 262–263.

[B31] Mancebo O Monzón CM Bulman GM (2001) *Haematobia irritans*: una actualización de diez años de su introducción en Argentina. Veterinaria (Argentina) 43: 34–46.

[B32] Manso F Lifschitz E (1986) Chromosome polymorphism in a population of *Ceratitis capitata*. II International Symposium on Fruit Flies. Creta, Amsterdam, 159–165.

[B33] Metz CW (1916) Chromosome studies of the Diptera. II. The paired association of chromosomes in the Diptera and its significance. Journal of Experimental Zoology 21: 213–280. doi: 10.1002/jez.1400210204

[B34] Oliveira MT Barau J Junqueira AC Feijao PC Rosa AC Abreu C Azeredo-Espin AM Lessinger A (2008) Structure and evolution of the mitochondrial genomes of *Haematobia irritans* and *Stomoxys calcitrans*: the Muscidae (Diptera: Calyptrata) perspective. Molecular and Phylogenetic Evolution 48: 850–857. doi: 10.1016/j.ympev.2008.05.022 10.1016/j.ympev.2008.05.022 18621550

[B35] Palmer WB Bay D (1981) A review of the economic importance of the horn fly, *Haematobia irritans irritans* (L.). Protection Ecology 3: 237–244.

[B36] Parise P Avancini R Recco-Pimentel S (1996) Karyotypic characterization of *Muscina stabulans* (Fallen) (Diptera: Muscidae) using conventional staining, silver staining and C-banding. Caryologia 49: 13–20. doi: 10.1080/00087114.1996.10797345

[B37] Parise P Avancini R (2007) Comparative cytogenetic study in Muscidae flies. Brazilian Journal of Biology 67: 945–950. doi: 10.1590/S1519-69842007000500020 10.1590/s1519-69842007000500020 18278364

[B38] Roderick G (1996) Geographic structure of insect population: gene flow, phylogenography, and their uses. Annual Review of Entomology 41: 325–352. doi: 10.1146/annurev.en.41.010196.001545 10.1146/annurev.en.41.010196.001545 15012332

[B39] SAS Institute Inc. (2013) Installing SAS Online Doc, Version 9.2, HTML Format, SAS Institute Inc., Cary, North Carolina.

[B40] Sheppard DC Torres PP (1998) Onset of resistance to fenvalerate, a pyrethroid insecticide in Argentina horn flies (Diptera: Muscidae). Journal of Medical Entomology 35: 175–176. 9538580 10.1093/jmedent/35.2.175

[B41] Silva A Jander G Samaniego H Ramsey J Figueroa C (2012) Insecticide resistance mechanisms in the green peach aphid *Myzus persicae* (Hemiptera: Aphididae) I: a transcriptomic survey. PLoS ONE 7(6): . doi: 10.1371/journal.pone.0036366 10.1371/journal.pone.0036366 PMC3369866 22685538

[B42] Stevens NM (1908) A study of the germ cells of certain Diptera with reference to the heterochromosomes and the phenomena of synapsis. Journal of Experimental Zoology 5: 359–374. doi: 10.1002/jez.1400050304

[B43] Tarelli GJ (2004) Moscas de los cuernos *Haematobia irritans* (L). Biologia, Comportamiento y Control. Editorial Hemisfério Sur. Buenos Aires, Argentina, 57 pp.

[B44] Torres PP Balbi A Sheppard DC Prieto OH Nuñez JL (1996) Resistencia a la mosca de los cuernos *Haematobia irritans* (L. 1758) al fenvalerato en la provincia de Corrientes. Revista de Medicina Veterinaria (Buenos Aires) 77: 136–140.

[B45] Valério JR Guimarães JG (1983) Sobre a ocorrência de uma nova praga, *Haematobia irritans* (L.) (Díptera: Muscidae), no Brasil. Revista Brasilera de Zoologia 1: 417–418.

[B46] Williams JD Sutherst RW Maymald GF Peterbridge CP (1985) The Southward spread of buffalo fly (*Haematobia irritans*) in eastern Australia and its survival through a severe winter. Australian Veterinary Journal 62: 367–369. doi: 10.1111/j.1751-0813.1985.tb14210.x 3834900 10.1111/j.1751-0813.1985.tb14210.x

